# Indications of a Scarring Effect of Sickness Absence Periods in a Cohort of Higher Educated Self-Employed

**DOI:** 10.1371/journal.pone.0156025

**Published:** 2016-05-23

**Authors:** Liesbeth E. C. Wijnvoord, Sandra Brouwer, Jan Buitenhuis, Jac J. L. van der Klink, Michiel R. de Boer

**Affiliations:** 1 Department of Health Sciences, Community and Occupational Medicine, University Medical Center Groningen, University of Groningen, Groningen, The Netherlands; 2 Medical Department Movir Insurance, Nieuwegein, The Netherlands; 3 Medical Department Univé Insurance, Assen, The Netherlands; 4 Department of Health Sciences and the EMGO Institute for Health and Care Research, Faculty of Earth and Life Sciences, VU University, Amsterdam, The Netherlands; 5 Dutch Academic Center for Insurance Medicine (DACIM), Groningen, The Netherlands; Catholic University of Sacred Heart of Rome, ITALY

## Abstract

**Objectives:**

Little is known regarding incidence and recurrence of sickness absence in self-employed. The primary aim of this study was to evaluate the influence of the number of prior episodes of sickness absence on the risk of subsequent periods of sickness absence in higher educated self-employed.

**Methods:**

In a historic register study based on the files of a Dutch private disability insurance company all sickness absence periods of 30 days or more were analysed.

**Results:**

A total of 15,868 insured persons contributed 141,188 person years to the study. In total, 5608 periods of sickness absence occurred during follow-up. The hazard of experiencing a new period of sickness absence increased with every previous period, ranging from a hazard ratio of 2.83 in case of one previous period of sickness absence to a hazard ratio of 6.72 in case of four previous periods. This effect was found for both men and women and for all diagnostic categories of the first period of sickness absence.

**Conclusions:**

Our study shows that for all diagnostic categories the hazard of experiencing a recurrence of sickness absence is appreciably higher than for experiencing a first episode. This suggests that this increased hazard may be related to the occurrence of sickness absence itself rather than related to characteristics of the insured person or of the medical condition. These findings could indicate that sickness absence periods may have a scarring effect on the self-employed person experiencing the sickness absence.

## Introduction

Self-employed workers face large problems when sickness absent: not only does the continuity of their business suffer but also this group does not receive sick pay similar to what wage-earners do [1]. These aspects negatively influence the financial situation of the self-employed. Few studies address work disability in this economically important group of the workforce despite the fact that both in the EU and in the USA the self-employed form an important segment of the workforce (over 30 million self-employed in the European Union and nearly 15 million in the USA) [2,3].

The studies on sick leave in self-employed have mostly been concerned with the duration of benefit payment,[4–8] and with interventions to shorten sickness absence [9]. Little is known about the incidence of sickness absence in self-employed and about diagnostic categories of the disorders associated with these periods of sickness absence. One study on the occurrence of sickness absence in relation to diagnoses in agriculturally self-employed found musculo-skeletal disorders to be the most common cause of sickness absence.[10] However, because of the specific workload of this population this outcome might not be generalizable to other occupational groups of self-employed.

In addition, to our knowledge no studies on recurrences of sickness absence in the self-employed have been performed so far. In employees there is evidence that a history of sick leave increases the risk of experiencing a new episode of sick leave.[11–15] Variations in some variables related to sickness absence between the self-employed and employees have been found which makes it questionable whether these findings for predicting recurrences of sickness absence in employees can be extrapolated. [1] Earlier studies comparing employees and self-employed have described the self-employed as being in better health than employees [16] and having more work satisfaction [17, 18]. These groups also differ in work engagement, found to be higher in self-employed [19] and in coping strategies [20].

As indicated previously, there is a significant knowledge gap regarding incidence and recurrence of sickness absence in the large and still growing population of self-employed. Information on the incidence of sickness absence in self-employed is important to provide insight into the extent of this issue and as a first step towards further research. Knowledge about recurrences of sickness absence and possible underlying mechanisms is important to identify vulnerable groups and can contribute to effective preventive actions targeted at high-risk groups to avoid or shorten recurrences of sickness absence. Insight into their own possible vulnerability to sickness absence can help self-employed to take precautions to ensure business continuity and prevent financial problems. Therefore, the aim of the present study is to evaluate the incidence and recurrence of periods of sickness absence in a sample of self-employed. We especially focused on the influence of the number of prior periods of sick leave, on the risk of subsequent sickness absence, on recurrences in relation to the diagnosis of the first period of sickness absence, and on gender differences.

## Methods

### Study population and study design

In the Netherlands disability insurance plans do not cover the financial consequences of sickness absence for the self-employed. Therefore, this group of workers have to obtain private disability insurance. Information from private insurance files can be used to collect information regarding sickness absence and disability in this population.

The present study is based on a dynamic cohort of 15,868 applicants for private income replacement insurance at Movir, a company insuring only college and university educated self-employed, (legal professions, general practitioners, other medical doctors/specialists, dentists or orthodontists, paramedic professions, technical professions, financial services, pharmacists, veterinarians and midwives) in the Netherlands. All applicants were included who applied for a new insurance policy covering sickness absence and long-term disability with a deferment period of 30 days, i.e. the waiting period before any benefit is paid, and who were accepted for insurance coverage between January 1 1993 and January 1 2010. Furthermore, only those applicants whose insurance contract ran for at least 18 consecutive months were included. All applicants were full-time self-employed or depended on their economic activities in self-employment for the main proportion of their personal income.

Ethical approval was sought from the Medical Ethics Committee of the University Medical Centre Groningen, which advised that, according to Dutch law, ethical clearance was not required for this study. Dutch law allows the use of personal data for scientific purposes by insurance companies so it is not necessary to obtain specific informed consent. In this study, all data were anonymised.

### Measurements

#### Sickness absence periods

In this study, the outcome variable was periods of sickness absence. As the shortest possible deferment period for the insurance company studied is 30 days, only periods of sickness absence of 30 calendar days or more were included. For every self-employed accepted for insurance coverage, information was extracted from the insurance company files on whether or not they had suffered one or more periods of sickness absence of 30 days or more between the day of acceptance and July 1 2011. The date of the first and the last day of these sickness absence episodes were registered. If clients were not insured for the whole follow-up period until July 1 2011 data were right censored on the date the insurance policy ended.

Periods of sickness absence were defined as the periods that individuals were unable to perform their own work fully, with no distinction made between partial and total sickness absence. Return to work in this study was defined as claim closure with the insurance company, which not automatically coincides with actual work resumption. Benefits for normal pregnancies were excluded, however, pregnancy related sickness cases were included. The diagnoses and the duration of the sickness absence periods were judged by the insurance company physician, using medical information from treating physicians and self-reported data from the insured.

#### Causes of sickness absence

Sickness absence causes were coded in the files of the insurance company. The diagnoses were grouped into the following main categories: mental and behavioural disorders, cardiovascular disorders, musculoskeletal disorders, pregnancy-related disorders, neoplasms (in all organ systems), acute infectious disorders (of any tract) and “other causes of sickness absence”. Other causes include e.g. neurological disorders, endocrine disorders, disorders of the eye and ear, and skin disorders. Data on sickness absence periods were checked for inconsistencies. Also, overlapping or directly consecutive periods, with no days between periods, were combined. In these combined periods the cause that was the reason for most days of absence was coded as the cause of the total sickness absence period.

#### Socio-demographic characteristics

The following characteristics of the individuals were retrieved from the insurance company files: gender, date of birth (used to calculate age at the start of the follow-up period) and profession.

### Statistical analyses

In order to describe the sample, we calculated numbers and percentages for categorical and means and standard deviations (SD’s) for continuous variables in SPSS 19. The incidence density of any episode of sickness absence was calculated by dividing the number of insured persons with a first episode by the person-years of the population at risk for a first episode. In addition, the 95% confidence interval was calculated. The total density of sickness absence periods was calculated by dividing the total number of sickness absence periods by the total time at risk. It was taken into account that during a period of sickness absence a person is not at risk for a recurrence of sickness absence. Incidence densities and total densities were expressed per 1000 person years.

The statistical package R3.01 was used to examine the influence of the number of previous episodes on recurrences of sickness absence.[21] In these analyses, the Andersen-Gill extension of the Cox proportional hazards model was used to allow for the recurrent nature of the event studied, here periods of sickness absence.[22]

Firstly, hazard ratios according to the number of previous periods of sickness absence were calculated for the entire sample. As a sensitivity analysis we restricted the sample to persons without pregnancy-related sickness absence. Next we stratified analyses for males and females and for different age categories. In all these analyses, the hazard of a second, third, fourth and fifth occurrence of sickness absence was related to the hazard of a first occurrence of sickness absence. Subsequently, hazard ratios stratified for the major diagnostic categories of the first episode of sickness absence were calculated. In these analyses, samples were restricted to persons with a specific major diagnosis for the first episode and the persons without any episode of sickness absence. The number of episodes was limited to the third occurrence because for a number of diagnostic categories the incidence of a fourth occurrence was very low.

## Results

### Descriptives of the study population

Table 1 presents demographic variables of the applicants included in our study. A total of 15,868 insured persons contributed 141,188 person years to the study. Almost 60% of the sample was male and the mean age at the start of the follow-up was 35.09 years. The sample consisted of higher educated self-employed (legal professions, general practitioners, other medical doctors/specialists, dentists or orthodontists, paramedic professions, technical professions, financial services, pharmacists, veterinarians and midwives, data not shown).

**Table 1:** Descriptives of the sample N = 15,868.

	N (%)	0 periods of SA	1 period SA	2 periods SA	3 or more periods SA
**Gender**					
**Men**	9459(59.6%)	7580 (80.2%)	1363 (14.4%)	358 (3.8%)	158 (1.7%)
**Women**	6409(40.4%)	4500 (70.2%)	1241 (19.4%)	409 (6.4%)	259 (4.0%)
**Age at start follow up Mean (SD)**	35.09(6.13)	34.85 (6.03)	35.93 (6.34)	35.92 (6.39)	35.20 (6.48)
**Median follow up in years (SD)**	8.38 (5.00)	6.83 (4.82)	12.66 (4.55)	13.49 (3.71)	13.63 (2.94)

In total, 5608 periods of sickness absence occurred during the follow-up. In our sample 12,080 individuals experienced no periods of sick leave. Incidence density of any sickness absence was 32.64 (95% CI 31.61–33.69) sickness absence periods per1000 person years, total density of sickness absence in our sample was 42.10 per 1000 person years. Women experienced a higher frequency of sickness absence than men. Fourteen persons died during follow-up.

### Sickness absence periods

Table 2 presents the characteristics of the periods of sickness absence according to major diagnostic categories. Additional information on other diagnostic categories can be found in S1 Appendix (Incidence density of sickness absence according to detailed diagnostic categories). Musculoskeletal disorders were by far the most frequent cause of sickness absence in our sample, followed by mental and behavioural disorders and sickness absence related to pregnancy and childbirth. Duration of the periods of sickness absence differed markedly, with periods of sickness absence caused by respiratory disorders and sickness absence related to appendicitis and inguinal herniation being among the shortest and duration of sickness absence for neoplasms and mental and behavioural disorders the longest. The exclusively female diagnostic categories of pregnancy and childbirth related disorders and other female genitourinary disorders accounted for relatively short periods as well. Chronic illnesses such as Parkinson’s disease and multiple sclerosis, as well as diabetes and whiplash-associated disorders, constituted the longest periods of sickness absence.

**Table 2:** Incidence density of sickness absence according to major diagnostic categories of the first episode.

Cause	N (%)	Incident period	Incidence density^a^ (95% CI)	Total density ^b^	Median duration Sickness absence periods (days)
**No sickness absence**	12080	n/a	n/a	n/a	n/a
**Musculoskeletal disorders**	2036 (36.3)	1373	11.829 (11.220–12.472)	15.284	102
**Mental and behavioural disorders**	1335 (23.8)	906	7.805 (7.313–8.330)	10.022	328
**Cardiovascular diseases**	223 (4.0)	150	1.292 (1.101–1.516)	1.674	231
**Pregnancy and childbirth related**	568 (10.1)	397	3.420 (3.010–3.774)	4.264	118
**Infectious disorders**	306 (5.5)	209	1.801 (1.573–2.062)	2.297	107.5
**Neoplasms**	233 (4.2)	166	1.430 (1.228–1.665)	1.749	332

n/a = not applicable

^a^. Incidence density = incident periods/1000 person-years at risk

^b^. Total density = total periods/ 1000 person-years at risk

For all causes of sickness absence we found that hazard rates of experiencing another period of sickness absence increased with the number of previous periods of sickness absence (see table 3). HR’s and CI’s were very similar in the sensitivity analysis restricted to persons without pregnancy-related sickness absence (data not shown). The same trend was observed for males and females separately.

**Table 3:** Hazard ratio according to number of previous periods of sickness absence regardless of cause for the total sample and stratified for gender.

	Total sample			Men			Women		
	n	HR	95% CI	n	HR	95% CI	n	HR	95% CI
**1 previous period**	3788	2.83	2.64–3.03	1879	2.88	2.59–3.19	1909	2.37	2.16–2.60
**2 previous periods**	1184	4.76	4.28–5.29	516	4.98	4.21–5.89	668	3.70	3.22–4.25
**3 previous periods**	417	5.77	4.87–6.85	158	6.77	5.12–8.96	259	4.22	3.40–5.25
**4 previous periods**	145	6.72	5.04–8.94	53	6.55	4.07–10.54	92	5.55	3.88–7.93

When stratifying for major diagnostic categories in first episodes of sickness absence a higher hazard rate was found for experiencing a second episode of sickness absence, regardless of diagnosis. For all causes of sickness absence except for cardiovascular disorders and neoplasms, we found that hazard rates of experiencing another period of sickness absence increased with the number of previous periods of sickness absence (see table 4).

**Table 4:** Hazard ratios stratified for diagnostic categories of first episodes of sickness absence related to hazard ratio for experiencing a recurrent episode of sickness absence.

Diagnosis first episode of sickness absence	n	HR experiencing second episode	95% CI	HR experiencing third episode	95% CI
**Musculoskeletal disorders**	1373	6.04	5.38–6.77	11.78	9.83–14.11
**Mental and behavioural disorders**	906	8.61	7.37–10.06	16.08	12.64–20.44
**Cardiovascular disorders**	150	48.26	5.93–12.50	35.12	15.30–80.63
**Pregnancy related disorders**	397	34.12	28.22–41.26	58.02	44.08–76.36
**Infectious disorders**	209	55.45	42.59–72.18	97.33	67.77–139.79
**Neoplasms**	166	58.96	41.28–84.22	37.72	17.35–82.01

## Discussion

The results of this study in higher educated self-employed showed that the hazard of experiencing a new period of sickness absence increased with every previous period. This effect was found for both sexes and also for most diagnostic categories of the first period of sickness absence. Musculoskeletal disorders and mental and behavioural disorders were the most frequent causes of long-term sickness absence. Locomotor disorders were more frequent, but mental disorders led to longer duration of sickness absence. This trend is in accordance with studies in employees regarding prevalence of causes of sickness absence.[23, 24] Our study found lower incidence densities, which is probably caused by only including longer periods of sickness absence.

Our most important finding was that for all causes of sickness absence combined the hazard rates of experiencing another period of sickness absence increased with the number of previous periods of sickness absence. These results are in line with the findings of other studies in employees.[15, 25, 26] However, the hazard rates found in our study are slightly higher than in these studies, which is probably caused by a lower incidence density in our sample, causing a higher hazard rate for recurrences. Although the confidence intervals of the consecutive categories did show overlap and increases levelled off after the third occurrence of sickness absence, the general trend in hazard ratios was clear. This may indicate that the hazard of experiencing a new period of sickness absence probably increases steadily and does not show sharp increments with every new period of sickness absence.

Individuals who experience multiple periods of sickness absence while the hazard remains constant regardless of the number of previous periods, may have a pre-existing vulnerability that does not change over time. This can sometimes be explained by persisting problems in work or private life.[15, 24, 27, 28] Hazard rates increasing proportionally to the number of previous periods of sickness absence can be reflective of the course of the underlying disease, or may point to a “scarring effect” of the sickness absence period itself as is described for depressive episodes.[29] The results found in our study seem to show a steady increase of hazard rates, thus providing support for one of the latter explanations.

Stratifying for major diagnostic categories in first episodes of sickness absence, an increase in hazard rates for experiencing a recurrent episode of sickness absence was found for all diagnoses, except for third episodes related to first episodes of sickness absence caused by cardiovascular disease and neoplasms. For most categories, the absolute value of the HR’s showed marked variation, which was caused by the fact that incidences (of first occurrences) differed, but the HR of experiencing a third occurrence was a factor 1.5–2 times higher than the HR of experiencing a second occurrence. This indicates that for all major diagnostic categories a similar effect of prior sickness absence periods was found. Although it is difficult to distinguish between the scarring effect and the course of the underlying disease as explanations for increasing hazard rates, these findings might suggest that a common underlying mechanism affects all individuals experiencing sickness absence, regardless of the diagnosis causing their first period of sickness absence. The nature of our dataset does not allow us to draw definite conclusions on possible underlying mechanisms. However, as this effect occurred in all diagnostic categories it is highly unlikely this must solely be attributed to characteristics of the medical condition (e.g. relapsing remitting conditions like migraine, or chronic conditions like diabetes mellitus).

### Strengths and limitations

The use of information from insurance company files can be considered as one of the strengths of this study. By this means, objective data on the self-employed were obtained for which there are few other sources, and recall-bias was prevented by using these files and not self-reported data.

Periods of sickness absence shorter than 30 days, mostly related to minor ailments, were not included in our study. Our data only included longer periods of sickness absence, restricting our study to more severe disorders. Longer sickness absence is an indication of a more serious health problem, which may be recurrent in nature. Recurrent short sickness absence, known to be indicative of dissatisfaction with work, is not represented in our dataset. However, self-employed may not resort to this behaviour of avoiding work in case of dissatisfaction with working circumstances. It can be assumed that the incidence densities and total densities found in this study underestimate sickness absence rates and that this probably influences hazard rates for recurrences as well. The exclusion of periods shorter than 30 days inflates the median duration of sickness absence in the present study.

Although the study was conducted in a large sample, diagnostic categories had to be combined in order to have sufficiently large groups of individuals. Self-employed with more than four recurrences of sickness absence were rare, rendering it more difficult to draw conclusions on this group. Another limitation concerns lack of data regarding comorbidity, severity of the disorder and of change of diagnosis during the sickness absence period, as only the main diagnosis was coded reliably in the files from the insurance company. Also, for this study we combined overlapping and directly consecutive periods of sickness absence according to the main diagnosis contributing most sickness days. Comorbidities are known to lengthen the duration of sickness absence periods.[30] Finally, as the sample in our study was specific, i.e. higher educated self-employed, a last limitation concerns the generalisablity of our results. Therefore some caution must be applied as to whether our findings are transferable to other populations of self-employed.

### Conclusions and implications

In conclusion, in this sample of higher educated self-employed it was found that the hazard of experiencing a new period of sickness absence increased with every previous period. These findings are important to recognize vulnerable groups of self-employed in order to develop strategies to prevent health problems and sickness absence. This effect was found for all diagnostic categories of the first period of sickness absence, suggesting that this may be related to the experience of sickness absence itself. However based on our data we cannot rule out other underlying mechanisms such as severity of disease.

Knowledge about incidence and recurrence of periods of sickness absence is important to identify vulnerable groups. Our study shows that for all diagnostic categories the hazard of experiencing a recurrence of sickness absence is appreciably higher than for experiencing a first episode. Developing interventions targeted at those self-employed who have already experienced one or more periods of sickness absence may therefore be more cost-effective than targeting interventions at the population as a whole. For self-employed, the awareness of their vulnerability for recurrence of sickness absence may be relevant as well and could be an additional motivation to make adjustments in their working conditions or in life style factors. The findings that the hazard of experiencing a new episode of sickness absence increases with the number of previous episodes and that this holds true for all major diagnostic categories suggests that sickness absence periods may have a scarring effect on the person experiencing the sickness absence. Further research is needed to gain insight in the nature of this effect.

## Supporting Information

S1 Appendix Incidence density of sickness absence according to detailed diagnostic categories. n/a = not applicable. a. Incidence density = incident periods/1000 person-years at risk. b. Total density = total periods/ 1000 person-years at risk.
(DOCX)
