# Supplementary material for: Indications of a Scarring Effect of Sickness Absence Periods in a Cohort of Higher Educated Self-Employed
Source: PLoS One. 2016 May 23;11(5):e0156025. doi: 10.1371/journal.pone.0156025 (PMC4877097; doi:10.1371/journal.pone.0156025)
Supplement: S1 Appendix — n/a = not applicable. a. Incidence density = incident periods/1000 person-years at risk. b. Total density = total periods/ 1000 person-years at risk. (DOCX) [file pone.0156025.s001.docx]

**Supporting information**

**S1 Appendix 1**.

Incidence density of sickness absence according to detailed diagnostic categories.

| **Cause** | **N (%)** | **Incident period** | **Incidence density^a^ (95% CI)** | **Total density ^b^** | **Median duration**  **Sickness absence periods (days)** |
| --- | --- | --- | --- | --- | --- |
| **No sickness absence** | 12080 | n/a | n/a | n/a | n/a |
| **Musculoskeletal disorders** | 2036 (36.3) | 1373 | 11.829 (11.220-12.472) | 15.284 | 102 |
| **Mental and behavioural disorders** | 1335 (23.8) | 906 | 7.805 (7.313-8.330) | 10.022 | 328 |
| **Pregnancy and childbirth related** | 568 (10.1) | 397 | 3.420 (3.010-3.774) | 4.264 | 118 |
| **Other female genitourinary disorders** | 101 (1.8) | 73 | 0.629 (0.500-0.791) | 0.758 | 73 |
| **Neoplasms** | 233 (4.2) | 166 | 1.430 (1.228-1.665) | 1.749 | 332 |
| **Cardiovascular diseases** | 223 (4.0) | 150 | 1.292 (1.101-1.516) | 1.674 | 231 |
| **(Acute) infectious diseases** | 306 (5.5) | 209 | 1.801 (1.573-2.062) | 2.297 | 107.5 |
| **Wounds/accidents** | 44 (0.8) | 30 | 0.258 (0.180-0.369) | 0.330 | 147.5 |
| **Respiratory disorders** | 80 (1.4) | 42 | 0.362 (0.268-0.490) | 0.601 | 85 |
| **Disorders of the eye** | 57 (1.0) | 35 | 0.302 (0.217-0.421) | 0.428 | 199 |
| **Ear/vestibular disorders** | 42 (0.7) | 28 | 0.241 (0.166-0.349) | 0.315 | 155.5 |
| **Skin disorders** | 33 (0.6) | 23 | 0.198 (0.132-0.298) | 0.248 | 96 |
| **Neurological disorders** | 30 (0.5) | 18 | 0.258 (0.163-0.409) | 0.225 | 1133 |
| **Headaches/migraines** | 19 (0.3) | 14 | 0.121 (0.072-0.204) | 0.143 | 135 |
| **Other disorders peripheral nerve system** | 64 (1.1) | 54 | 0.465 (0.356-0.607) | 0.480 | 198.5 |
| **Whiplash/chronic fatigue/other medically unexplained symptoms** | 18 (0.3) | 9 | 0.078 (0.041-0.150) | 0.135 | 509.5 |
| **Diabetes mellitus** | 8 (0.1) | 6 | 0.052 (0.023-0.116) | 0.060 | 3090 |
| **Other endocrine and immune disorders** | 39 (0.7) | 26 | 0.224 (0.153-0.329) | 0.293 | 144 |
| **Gallbladder/inguinal hernia/appendix** | 59 (1.0) | 45 | 0.388 (0.290-0.520) | 0.443 | 58 |
| **Inflammatory bowel disease** | 28 (0.5) | 11 | 0.095 (0.053-0.172) | 0.210 | 149 |
| **Other gastrointestinal disorders** | 95 (1.7) | 64 | 0.551 (0.431-0.704) | 0.713 | 113 |
| **Kidneys/urinary tract** | 38 (0.7) | 26 | 0.224 (0.153-0.329) | 0.285 | 139.5 |
| **Other causes** | 152 (2.7) | 83 | 0.715 (0.577-0.887) | 1.141 | 122 |

n/a= not applicable

1. Incidence density = incident periods/1000 person-years at risk
2. Total density = total periods/ 1000 person-years at risk
